# Supplementary material for: Interactive effects of developmental and adult nutrition on lifespan and fecundity in a genetically diverse Drosophila population
Source: PLoS One. 2025 Oct 10;20(10):e0334341. doi: 10.1371/journal.pone.0334341 (PMC12513662; doi:10.1371/journal.pone.0334341)
Supplement: S1 File — (DOCX) [file pone.0334341.s001.docx]

**Additional tables**

**Table 1**: **Summary of survival and per female fecundity in each larval-adult treatment groups.**

| Regime | Survival | All | | | Female | | |  | Male | | |
| --- | --- | --- | --- | --- | --- | --- | --- | --- | --- | --- | --- |
|  | (%) | N | N_ev_ | Days (CI) | N | N_ev_ | Days (CI) | Eggs±se* | N | N_ev_ | Days (CI) |
| HH | 90 | 674 | 638 | 10(10-12) | 426 | 407 | 12(10-15) | 6.5±1.6 | 248 | 231 | 8(8-12) |
|  | 50 |  |  | 31(29-33) |  |  | 26(26-29) | 3.9±0.6 |  |  | 38(36-40) |
|  | 10 |  |  | 61(57-66) |  |  | 57(57-61) | 1.7±0.4 |  |  | 68(64-78) |
|  |  |  |  |  |  |  |  |  |  |  |  |
| HL | 90 | 721 | 676 | 12(10-15) | 393 | 370 | 15(10-17) | 6.8±1.1 | 328 | 306 | 10(8-19) |
|  | 50 |  |  | 38(36-40) |  |  | 36(36-40) | 8.5±2.1 |  |  | 38(36-40) |
|  | 10 |  |  | 71(68-78) |  |  | 64(61-68) | 1.0±0.2 |  |  | 87(85-92) |
|  |  |  |  |  |  |  |  |  |  |  |  |
| LH | 90 | 937 | 856 | 12(10-15) | 625 | 585 | 12(12-15) | 12.1±2.6 | 312 | 271 | 10(8-17) |
|  | 50 |  |  | 36(33-36) |  |  | 31(29-33) | 5.1±1.1 |  |  | 38(38-40) |
|  | 10 |  |  | 68(64-71) |  |  | 64(59-66) | 1.0±0.2 |  |  | 78(71-78) |
|  |  |  |  |  |  |  |  |  |  |  |  |
| LL | 90 | 800 | 742 | 12(12-15) | 510 | 481 | 12(12-15) | 6.8±0.6 | 290 | 261 | 12(10-19) |
|  | 50 |  |  | 38(36-40) |  |  | 36(33-38) | 12.6±2.4 |  |  | 43(40-47) |
|  | 10 |  |  | 68(66-73) |  |  | 64(59-66) | 1.2±0.2 |  |  | 87(78-92) |

N is the number of individuals in a treatment, N_ev_ is event count (i.e., deaths), Survival is the number of days for that treatment group to reach 90%, 50% and 10% survival (i.e., 10%, 50%, 90% mortality), CI, confidence intervals around survival estimates, se, standard error of mean eggs. *Mean number of eggs per female per 3 hour-laying period.

**Table 2: Cox model selection. Best models are bold-faced.**

| Models | k | AICc | ΔAICc | Wt |
| --- | --- | --- | --- | --- |
| 1. LIFESPAN | | | | |
| 1. All flies (*n* = 3134; events = 2912) | | | | |
| **s(Sex) + C** | **7** | **37446.42** | **0.00** | **0.28** |
| **L_A + s(Sex) + C** | **7** | **37446.42** | **0.00** | **0.28** |
| **L_A + s(Sex) + C + L_A*C** | **7** | **37446.40** | **0.00** | **0.28** |
| L_A + s(Sex) + C + L_A*s(Sex) | 10 | 37449.40 | 2.98 | 0.06 |
| L_A + s(Sex) + C + L_A*s(Sex) + L_A*C | 10 | 37449.40 | 2.98 | 0.06 |
| L_A + s(Sex) | 3 | 37452.39 | 5.97 | 0.01 |
| L_A + s(Sex) + L_A*Sex | 6 | 37455.19 | 8.77 | 0.00 |
| s(Sex) | 0 | 37478.34 | 31.92 | 0.00 |
| C | 7 | 41257.91 | 3811.49 | 0.00 |
| L_A + C | 7 | 41257.91 | 3811.49 | 0.00 |
| L_A + C + L_A*C | 7 | 41257.91 | 3811.49 | 0.00 |
| L_A | 3 | 41262.36 | 3815.95 | 0.00 |
| I | 0 | 41297.91 | 3851.50 | 0.00 |
|  |  |  |  |  |
| 2) Females only (*n* = 1956; events = 1843) | | | | |
| **L_A + C** | **7** | **24430.26** | **0.00** | **0.29** |
| **L_A + C + L_A*C** | **7** | **24430.26** | **0.00** | **0.29** |
| **C** | **7** | **24430.26** | **0.00** | **0.29** |
| **L_A** | **3** | **24431.65** | **1.39** | **0.14** |
| I | 0 | 24440.62 | 10.36 | 0 |
|  |  |  |  |  |
| 3) Males only (*n* = 1178; events = 1069) | | | | |
| **L_A** | **3** | **13023.54** | **0.00** | **0.63** |
| C | 7 | 13026.78 | 3.24 | 0.12 |
| L_A + C | 7 | 13026.78 | 3.24 | 0.12 |
| L_A + C + L_A*C | 7 | 13026.78 | 3.24 | 0.12 |
| I | 0 | 13037.72 | 14.18 | 0 |
|  |  |  |  |  |
| B. FECUNDITY |  |  |  |  |
| **L_A + L_A*Age +C** | **13** | **3489.9** | **0.00** | **0.44** |
| **L_A + L_A*Age +C + L_A*C** | **13** | **3489.9** | **0.00** | **0.44** |
| L_A*Age + L_A*C | 9 | 3492.4 | 2.50 | 0.13 |
| L_A | 10 | 3510.5 | 20.57 | 0.00 |
| L_A + L_A*Age | 10 | 3510.5 | 20.57 | 0.00 |
| L_A + L_A*Age + L_A + L_A*Age | 10 | 3510.5 | 20.57 | 0.00 |
|  |  |  |  |  |

**Table 3: Detailed model results. For averaged models, we interpret conditional averages.**

|  | Estimate | Std. error | z | Pr(>\|z\|) | Significance level |
| --- | --- | --- | --- | --- | --- |
| **Lifespan all flies: Model-averaged coefficients** | | | | |  |
| **Full average** | | | | |  |
| cageIDH_H2 | 0.01647 | 0.07973 | 0.207 | 0.8364 |  |
| cageIDH_L1 | -0.15564 | 0.08730 | 1.783 | 0.0746 | . |
| cageIDH_L2 | -0.33291 | 0.07691 | 4.328 | 1.5e-05 | *** |
| cageIDL_H1 | -0.07742 | 0.08243 | 0.939 | 0.3476 |  |
| cageIDL_H2 | -0.14006 | 0.07306 | 1.917 | 0.0552 | . |
| cageIDL_L1 | -0.38949 | 0.08743 | 4.455 | 8.4e-06 | *** |
| cageIDL_L2 | -0.16659 | 0.07441 | 2.239 | 0.0252 | * |
|  |  |  |  |  |  |
| **Model-averaged coefficients: female lifespan** | | | | |  |
| **Full average** | | | | |  |
| cageIDH_H2 | 0.002111 | 0.092872 | 0.023 | 0.9819 |  |
| cageIDH_L1 | -0.107456 | 0.119383 | 0.900 | 0.3681 |  |
| cageIDH_L2 | -0.274199 | 0.144394 | 1.899 | 0.0576 | . |
| cageIDL_H1 | -0.084467 | 0.101305 | 0.834 | 0.4044 |  |
| cageIDL_H2 | -0.120907 | 0.098277 | 1.230 | 0.2186 |  |
| cageIDL_L1 | -0.303821 | 0.161252 | 1.884 | 0.0595 | . |
| cageIDL_L2 | -0.095970 | 0.095261 | 1.007 | 0.3137 |  |
| larv_adultH_L | -0.088002 | 0.131206 | 0.671 | 0.5024 |  |
| larv_adultL_H | -0.042469 | 0.070713 | 0.601 | 0.5481 |  |
| larv_adultL_L | -0.065284 | 0.100195 | 0.652 | 0.5147 |  |
| **Honditional average female** | | | | |  |
| cageIDH_H2 | 0.002463 | 0.100308 | 0.025 | 0.980412 |  |
| cageIDH_L1 | -0.125364 | 0.119927 | 1.045 | 0.295866 |  |
| cageIDH_L2 | -0.319896 | 0.098519 | 3.247 | 0.001166 | ** |
| cageIDL_H1 | -0.098544 | 0.102888 | 0.958 | 0.338174 |  |
| cageIDL_H2 | -0.141058 | 0.091792 | 1.537 | 0.124363 |  |
| cageIDL_L1 | -0.354455 | 0.111302 | 3.185 | 0.001449 | ** |
| cageIDL_L2 | -0.111965 | 0.093789 | 1.194 | 0.232557 |  |
| larv_adultH_L | -0.264016 | 0.071947 | 3.670 | 0.000243 | *** |
| larv_adultL_H | -0.127411 | 0.064645 | 1.971 | 0.048730 | * |
| larv_adultL_L | -0.195860 | 0.067407 | 2.906 | 0.003665 | ** |
|  |  |  |  |  |  |
| **Males lifespan** | | | | |  |
| **Top model** |  |  |  |  |  |
| larv_adultH_L | -0.31340 | 0.08932 | -3.509 | 0.00045 | *** |
| larv_adultL_H | -0.11852 | 0.08960 | -1.323 | 0.18588 |  |
| larv_adultL_L | -0.36169 | 0.09150 | -3.953 | 7.72e-05 | *** |
|  |  |  |  |  |  |
| **Female fecundity: age-specific number of eggs.** Note: empty terms have been omitted but can be retrieved by running the script (see manuscript for link to GitHub repository) | | | | | |
| Full average |  | Adjusted |  |  |  |
| (Intercept) | 129.6244 | 36.9908 | 3.504 | 0.000458 | *** |
| age | -2.4505 | 0.7461 | 3.284 | 0.001022 | ** |
| cageH_H2 | 39.6561 | 33.1744 | 1.195 | 0.231938 |  |
| cageH_L1 | -13.9415 | 51.5091 | 0.271 | 0.786652 |  |
| cageH_L2 | 10.6005 | 51.5091 | 0.206 | 0.836948 |  |
| cageL_H1 | 235.6120 | 52.1254 | 4.520 | 6.20e-06 | *** |
| cageL_H2 | 319.3923 | 51.2138 | 6.236 | < 2e-16 | *** |
| cageL_L1 | 50.6508 | 51.5091 | 0.983 | 0.325442 |  |
| cageL_L2 | 98.6317 | 51.5091 | 1.915 | 0.055513 | . |
| age:laradH_L | 0.5133 | 1.0091 | 0.509 | 0.610987 |  |
| age:laradL_H | -4.0953 | 1.0178 | 4.024 | 5.73e-05 | *** |
| age:laradL_L | -0.6231 | 1.0091 | 0.617 | 0.536921 |  |
| **Conditional average** | | | | |  |
| (Intercept) | 129.6244 | 36.9908 | 3.504 | 0.000458 | *** |
| age | -2.4505 | 0.7461 | 3.284 | 0.001022 | ** |
| cageH_H2 | 39.6561 | 33.1744 | 1.195 | 0.231938 |  |
| cageH_L1 | -13.9415 | 51.5091 | 0.271 | 0.786652 |  |
| cageH_L2 | 10.6005 | 51.5091 | 0.206 | 0.836948 |  |
| cageL_H1 | 235.6120 | 52.1254 | 4.520 | 6.20e-06 | *** |
| cageL_H2 | 319.3923 | 50.9782 | 6.236 | < 2e-16 | *** |
| cageL_L1 | 50.6508 | 51.5091 | 0.983 | 0.325442 |  |
| cageL_L2 | 98.6317 | 51.5091 | 1.915 | 0.055513 | . |
| age:laradH_L | 0.5133 | 1.0091 | 0.509 | 0.610987 |  |
| age:laradL_H | -4.0953 | 1.0178 | 4.024 | 5.73e-05 | *** |
| age:laradL_L | -0.6231 | 1.0091 | 0.617 | 0.536921 |  |
|  |  |  |  |  |  |
| **Fecundity: per female per 3-hour oviposition period** | | | | |  |
| **Full average** |  |  |  |  |  |
| (Intercept) | 6.983732 | 1.361403 | 5.130 | 3e-07 | *** |
| age | -0.083712 | 0.026941 | 3.107 | 0.00189 | ** |
| laradH_L | 0.439817 | 1.318614 | 0.334 | 0.73872 |  |
| laradL_H | 3.442820 | 2.735720 | 1.258 | 0.20822 |  |
| laradL_L | 1.507011 | 1.661719 | 0.907 | 0.36446 |  |
| age:laradH_L | -0.006939 | 0.028523 | 0.243 | 0.80780 |  |
| age:laradL_H | -0.062376 | 0.052119 | 1.197 | 0.23138 |  |
| age:laradL_L | -0.019176 | 0.031158 | 0.615 | 0.53826 |  |
| **Conditional average** | | | | |  |
| (Intercept) | 6.98373 | 1.36140 | 5.130 | 3e-07 | *** |
| age | -0.08371 | 0.02694 | 3.107 | 0.00189 | ** |
| laradH_L | 0.65628 | 1.56603 | 0.419 | 0.67516 |  |
| laradL_H | 5.13730 | 1.56929 | 3.274 | 0.00106 | ** |
| laradL_H | 5.13730 | 1.56929 | 3.274 | 0.00106 | ** |
| laradL_L | 2.24873 | 1.56603 | 1.436 | 0.15102 |  |
| age:laradH_L | -0.01035 | 0.03433 | 0.302 | 0.76297 |  |
| age:laradL_H | -0.09308 | 0.03458 | 2.692 | 0.00711 | ** |
| age:laradL_L | -0.02861 | 0.03433 | 0.833 | 0.40456 |  |
