# Supplementary material for: Interactive effects of developmental and adult nutrition on lifespan and fecundity in a genetically diverse Drosophila population
Source: PLoS One. 2025 Oct 10;20(10):e0334341. doi: 10.1371/journal.pone.0334341 (PMC12513662; doi:10.1371/journal.pone.0334341)
Supplement: S2 File — (DOCX) [file pone.0334341.s002.docx]

**Additional figures**

**Figure S1: Kaplan-Meier plots of survival probabilities:**

*left*: [age, status] ~ larval diet + adult diet and

*right*: accounting for sex [age, status] ~ larval diet + adult diet + sex. HP, LP stand for high protein and low protein, respectively.





**Figure S2: Patterns of fecundity calculated from egg counts from 3 hours mating periods 3 times per week: a.** overall and **b.** time series of egg counts per female over lifetime. **c.** Relationship between lifespan and fecundity – patterns of fecundity across quartiles 1-4 of lifespan. Clearly, the timing and magnitude of peak reproductive output varies across diet exposures. HH, HL, LH, and LL stand for high-larval-high-adult, high-larval-low-adult, low-larval-high-adult, and low-larval-low-adult protein.
